# The Source Areas and Migratory Pathways of the Fall Armyworm *Spodoptera frugiperda* (Smith) in Sichuan Province, China

**DOI:** 10.3390/insects13100935

**Published:** 2022-10-16

**Authors:** Chunxian Jiang, Xueyan Zhang, Jiaqi Wu, Chuanhong Feng, Li Ma, Gao Hu, Qing Li

**Affiliations:** 1 College of Agronomy, Sichuan Agricultural University, Chengdu 611130, China; 2 College of Plant Protection, Nanjing Agricultural University, Nanjing 210095, China; 3 Plant Protection Station, Sichuan Provincial Department of Agriculture and Rural Affairs, Chengdu 610041, China

**Keywords:** *Spodoptera frugiperda*, source areas, migration trajectory, HYSPLIT, trajectory analysis

## Abstract

**Simple Summary:**

Identifying source areas and migratory pathways is very important for monitoring and providing an early warning of migratory pests. The newly invasive pest *Spodoptera frugiperda* (Smith), which is a serious threat to China’s food production, migrates along two migratory pathways in China: the eastern pathway and the western pathway. The Sichuan Province, located in Southwest China, is an important node along the western pathway of *S. frugiperda*. However, the source areas and migratory pathway of *S. frugiperda* in Sichuan are not clear. As a result, trajectory simulations were used to study the source areas and migratory pathways of *S. frugiperda* in Sichuan. The results showed that the source area of *S. frugiperda* in Sichuan was widely distributed. The source areas were located not only along the western national migration pathway but also along the eastern pathway. *S. frugiperda* migrated to Sichuan from the source areas via 6 potential pathways, 1 pathway into southwest Sichuan and 5 pathways into the Sichuan basin. This study can provide new information on the migratory pathways of *S. frugiperda* in China and can help with the monitoring and early warning of the presence of *S. frugiperda* in Sichuan and throughout all of China.

**Abstract:**

The Sichuan Province, located in Southwest China, is one of China’s main maize-producing areas, and is also an important node along the north-south migratory pathways that pests follow within China. After its invasion, the fall armyworm (FAW), *Spodoptera frugiperda* (Smith), was found in 70.81% of all counties in Sichuan. However, FAW source areas and their migratory pathways into Sichuan remain unclear. This study simulated FAW sources and their migratory pathways into Sichuan during 2020 and 2021 using the trajectory simulation platform HYSPLIT with flight behavior parameters. Additionally, the seasonal horizontal wind field was also analyzed with the meteorological graphics processing software GrADS. The results showed that sporadic FAW migration into Sichuan began in April. By May, FAWs were found in much of the Sichuan Basin and moved further north and west in June. Except for year-round breeding areas, FAW sources varied monthly and expanded northward and eastward. The source areas were concentrated in Yunnan, Guizhou, Chongqing, and Myanmar on the western pathway of national migration and also in Vietnam, Guangxi, and Hunan of the eastern pathway. At various times, parts of Sichuan have also served as sources for other parts of Sichuan. FAWs migrated to Sichuan from the source areas via 6 potential pathways, 1 pathway into southwest Sichuan and 5 pathways into the Sichuan basin. The southwestern airflow from the Bay of Bengal, the southeastern airflow controlled by the western Pacific subtropical high, and the local topographically influenced airflow could provide the airflow needed for FAW migration. This work provides new information that can assist the monitoring and warning of the presence of FAW and support integrated management strategies for this pest in Sichuan and throughout China.

## 1. Introduction

The fall armyworm (FAW), *Spodoptera frugiperda* (Smith), originating from tropical and subtropical areas of the Americas [1,2], is a global invasive migratory pest [3,4,5]. The biological and ecological characteristics of the FAW, such as a strong migratory capability, high fecundity, and polyphagous and ecological plasticity, are the main reasons for the invasions and outbreaks in its native and newly invaded areas [6]. Since 2016, FAWs have invaded and expanded rapidly from the Americas to Africa and Asia. FAWs invaded China for the first time at the end of 2018 and then spread rapidly. By 2020, the FAW had expanded to 1426 counties in 27 provinces (autonomous regions and municipalities) in China [7]. Maize is the primary crop damaged by the FAW in China [8,9]. The Ministry of Agriculture and Rural Development of China added FAWs to The list of First-class Crop Diseases and Pests in China [10].

**Figure 1:**
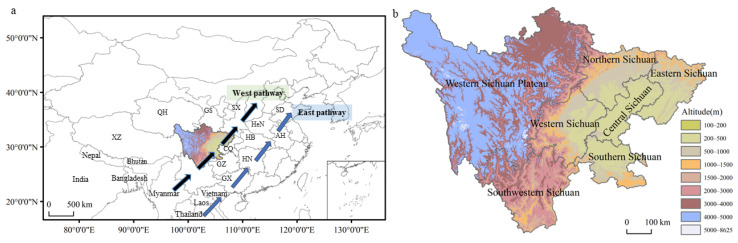
(**a**) The geographical location of Sichuan in China. Sichuan is on the western migratory pathway of FAWs. (**b**) Topography and divisions of Sichuan Province. Sichuan Province is divided into seven topographic regions: southwestern Sichuan, where FAWs can breed year-round in the subtropical areas of this region; western Sichuan Plateau, where FAWs occur sporadically as less than one generation; and the other five regions, i.e., southern Sichuan, western Sichuan, central Sichuan, northern Sichuan and eastern Sichuan, which compose the Sichuan Basin and its surroundings with hills and mountains. Two-letter codes refer to the different provinces of China (XZ: Tibetan Autonomous Region, QH: Qinghai Province, GS: Gansu Province, SX: Shannxi, HeN: Henan Province, HB: Hebei Province, AH: Anhui Province, HN: Hunan Province, GX: Guangxi Province, GZ: Guizhou Province, SD: Shandong Province).

Currently, FAWs can breed year-round in some areas of southwestern China and southern China and have already established a seasonal migration pattern in China [11,12]. The overseas source areas of FAWs in China are mainly located in the year-round breeding areas on the Indochina Peninsula, such as Myanmar, Thailand, Vietnam, and Laos. The domestic source areas are in the year-round breeding areas south of 28°0’0’’ N and the overwintering areas at 28°0’0’’ N~31°0’0’’ N in the southern and southwestern parts of the country [11,12,13,14]. FAWs migrate northward from their source areas along two pathways every year. The eastern pathway originates from Thailand, Laos, Vietnam, and southern China’s year-round breeding and overwintering areas. From there, FAWs gradually migrate northward into the Yangtze River Basin, the Huang-Huai Region, and areas north of the Yellow River, reaching as far as northeastern China. The western pathway originates from Myanmar and the year-round breeding and overwintering areas of the Yunnan Province, and it then passes through Guizhou and Sichuan and reaches the Shanxi, Shaanxi, and Gansu Provinces (Figure 1a) [11,15].

The Sichuan Province is located in southwestern China at the upper reach of the Yangtze River. The region has varied topography, that is, high in the west and low in the east. The main types of landforms in the province are plains, hills, mountains, and plateaus. In the middle of the Sichuan Province lies the Sichuan Basin, which is surrounded by hills and mountains. To the west of the basin is the West Sichuan Plateau, which comprises a transitional area leading into the Qinghai-Tibet Plateau [16,17]. The Sichuan Province can be divided into seven topographic regions (Figure 1b) and is also one of the main maize-growing areas in China, with more than 1.8 million hm^2^ used for growing maize each year. The diverse landforms and climate have created a self-contained insect migratory environment in Sichuan [18,19,20].

Moreover, the Sichuan Province is an important node for the north-south migration of migratory pests (e.g., *Sogatella furcifera* (Horváth)) within China [16]. After breeding in Sichuan, the offspring of migratory pests can migrate further northward and become insect sources in the north of Sichuan, even northern China [16,20,21]. Maxent model simulations showed that except for the Western Sichuan Plateau, most of Sichuan Province is suitable for FAWs, with the suitable area accounting for 57% of the total area of the Sichuan Province (unpublished data). In these areas, which are also the main maize-growing areas of Sichuan, FAWs occur for between one and six generations, with a maximum of seven generations [22]. On 8 May 2019, FAW larvae were found in maize fields in the town of Lizhou, in Xichang city, southwestern Sichuan. This was the first time that FAWs were found in Sichuan [23]. By 2020, FAWs had been found in 70.81% of all counties in the Sichuan Province (these statistical data were provided by the Department of Agriculture and Rural Affairs of Sichuan Province). FAWs can breed year-round in the subtropical areas of southwestern Sichuan and overwinter in some parts of southern Sichuan. However, FAWs primarily migrate from outside Sichuan [23]. Therefore, understanding the distribution of source areas and migration pathways is crucial for accurately monitoring and providing an early warning of migratory pests, especially for their effective control source areas [24]. Previous studies have shown that Sichuan was on the western migratory pathway of the FAW in China [11,15]. The author previously studied the migration of FAWs in Sichuan during the first year of invasion [23]. However, after invading China, FAWs bred year-round in the southwestern and southern provinces, including Sichuan. As a result, the current distribution of the FAW source areas and migratory pathways may now differ from those during the first year of invasion.

In this study, the FAW source areas and potential migratory pathways in Sichuan Province during 2020 and 2021 were modelled using trajectory simulations combined with the flight behavior parameters of the FAW. Additionally, the background weather conditions during the migration period were also analyzed. This study will help to improve FAW monitoring and early warning capabilities and provide information for integrated management strategies of this pest in Sichuan and throughout China.

## 2. Materials and Methods

### 2.1. Data Sources

Insect data: The FAW field survey data collected in the Sichuan Province from January to June in 2020 and 2021 were provided by the Plant Protection Station of Sichuan Province. These data included the dates of initial detection, host plants, and larval stages of FAWs in each county among the 7 regions. The occurrence of data in other areas, including other provinces in China and countries around China, came from The Plant Diseases and Pest Information issued by the National Agro-Tech Extension Service Center of China (NATESC) as well as the plant protection stations within each province. These data were used to either filter or verify the possible FAW source areas that were estimated by the trajectory approach.

Meteorological data: Final Analysis (FNL) data, took the form of a 6-hourly, global, 1-degree gridded meteorological dataset, from the National Centers for Environmental Prediction (NCEP) and the National Center for Atmospheric Research (NCAR) (https://rda.ucar.edu/datasets/ds083.2/index.html, accessed on 14 January 2022), were used as meteorological data in this study.

### 2.2. Migration Dynamics Analysis

Since the field survey data were mostly larval data, the migration dates of the FAW moths were estimated based on the first date of FAW detection, the first detection of the larval stage, and the duration of each stage from larva to adult [25,26,27]. When the larvae were in their 2nd, 3rd, 4th, 5th and 6th instar, the migration dates of moths were 4–5 d, 5–7 d, 7–9 d, 9–11 d, and 12–14 d from the date that the larvae were detected, respectively [23,26,27]. To explore the seasonal variability of FAW migration in Sichuan, the geographic coordinates and the estimated migration dates over every ten days within each region were aggregated, and the monthly FAW migration map from April to June in 2020 and 2021 was drawn using ArcGIS (version 10.4, Esri, California, USA).

### 2.3. Migration Trajectory Analysis

According to the FAW migration dates and geographical locations, a total of 17 representative sites from each region in 2020 and 22 representatives in 2021 were selected to analyze the migration trajectory of FAWs from April to June (Tables S1 and S2). The HYSPLIT (Hybrid Single-Particle Lagrangian Integrated Trajectory Model) online platform (https://www.ready.noaa.gov/HYSPLIT_traj.php, accessed on 11 February 2022), which was developed by the National Oceanic and Atmospheric Administration of the United States and the Australian Bureau of Meteorology, was used to simulate the FAW migration trajectories. This platform has been widely applied to simulate the migration trajectory of many seasonal migratory pests, such as *C.* *medinalis*, *S.* *furcifera* [16,28], and also FAW [4]. The simulation of this platform has been proven to have a high accuracy by the mark-recapture experiment of rice planthoppers [29]. Backward trajectories were simulated based on the following FAW flight characteristics and parameters: (1) FAWs fly downwind [30,31]; (2) FAWs mostly fly for three consecutive nights [11]; (3) FAWs fly at night, taking off at dusk and landing on the following dawn [32], and FAWs can fly continuously for 10 h every night [33,34]. According to the sunrise and sunset data in Sichuan, the takeoff time was set to 20:00 Beijing time (12:00 UTC), and the landing time was set to 6:00 Beijing time (22:00 UTC). The endpoints of the back trajectories of the first 10 h flight were set as the new starting points for the next 10 h flight on the following night. The third flight night was set the same way as the second flight. (4) Since the accurate flight height of the FAW was not clear, five initial heights of 500, 750, 1000, 1250, and 1500 m above ground level (AGL) were set to ensure capture of the most likely flight heights [4,5,35]. All the trajectory endpoints obtained were imported into ArcGIS 10.4, and valid endpoints were filtered based on the criteria pertaining to suitable hosts and FAWs with suitable stages. Then, the spatial distribution probability of valid trajectory endpoints was calculated and plotted by the spatial analysis module of ArcGIS. The steps were as follows: (1) define a cell grid of 0.5° × 0.5° to cover all valid endpoints; (2) calculate the number of valid endpoints in each cell grid; (3) calculate the endpoint probability of each grid for display on the map.

### 2.4. Meteorological Background Analysis

Since airflow is the main factor affecting the migration of FAWs [11,34], horizontal wind fields at 850 hPa during the migration periods were extracted from the Final Analysis (FNL) data using the meteorological graphics processing software GrADS 2.1 (http://cola.gmu.edu/grads, accessed on 15 October 2022), and the airflow that carried the FAWs was analyzed.

## 3. Results

### 3.1. Migration Dynamics of FAW in Sichuan

**Figure 2:**
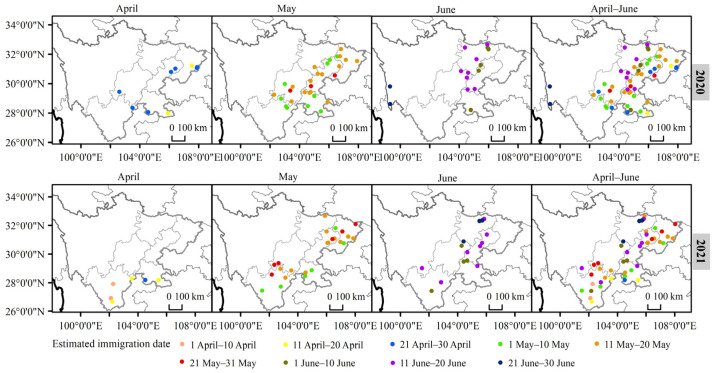
FAW migration dynamics in the Sichuan Province from April to June 2020 and 2021. The colored dots represent the sites where the migration of FAWs have been detected in different time periods. The year and time periods of migration are labeled on the top/right of each panel.

Field surveys indicated that the FAW reproduced year-round in the subtropical areas of southwestern Sichuan, such as Panzhihua and the southwestern part of Liangshan, with warm winters and average temperatures of up to 13.07 °C in January [22]. Maize was also grown there in winter. FAWs began to migrate into the Sichuan Basin sporadically in April and at a large scale in May. By May, FAWs were found in most areas of the Sichuan Basin. By June, the FAWs migrated further north and west. The westernmost extent of the FAW migration was the west of the Western Sichuan Plateau, near the boundary of the Western Sichuan Plateau, Tibet and Yunnan (Figure 2). The migration time of FAWs in 2021 was slightly later than that in 2020, especially for the time to migrate to eastern and central Sichuan, which was approximately 10 days later than the previous year (Figure 2).

### 3.2. Source Areas of FAW Migrating to Sichuan

**Figure 3:**
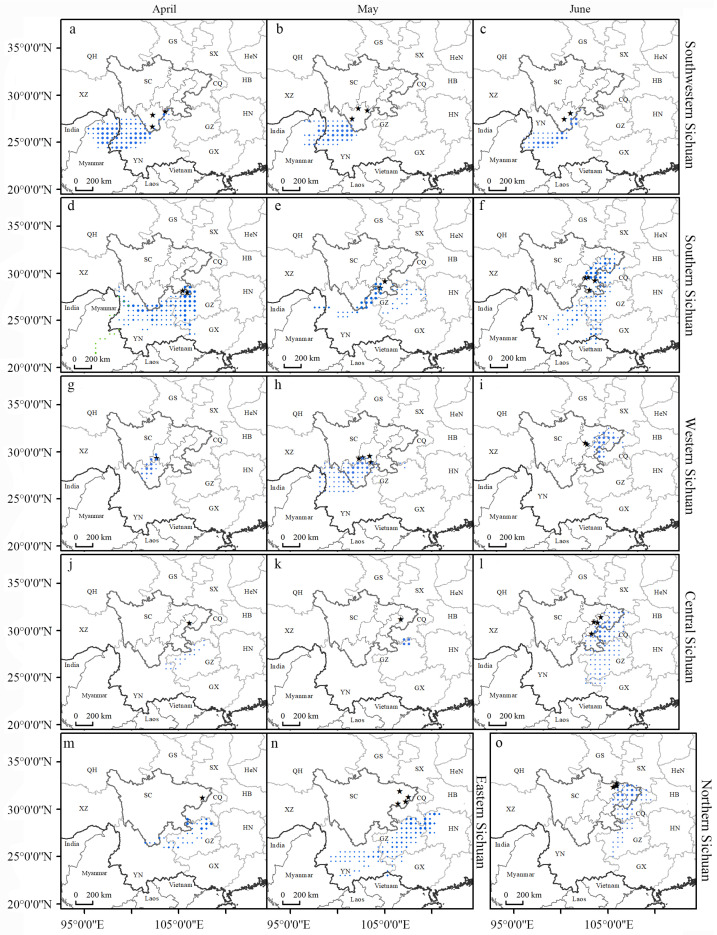
Distribution of endpoints for the FAW backward migration trajectories of the different regions of Sichuan Province from April to June. The black pentagrams are the starting points of the backward trajectories of each region. The region is labeled on the left of the panel, while the time periods of trajectories are labeled on the top. The distribution of dots indicates the distribution of source areas of FAW. The larger the dots are, the greater the distribution probability. The two-letter codes refer to the different provinces of China (SC: Sichuan Province, YN: Yunnan Province, GX: Guangxi Zhuang Autonomous Region, GZ: Guizhou Province, CQ: Municipality of Chongqing, HN: Hunan Province, HB: Hubei Province, HeN: Henan Province, SX: Shaanxi Province, GS: Gansu Province, XZ: Tibet Autonomous Region, QH: Qinghai Province). (**a**–**c**), Distribution of trajectory endpoints in southwestern Sichuan in April, May and June, respectively; (**d**–**f**), Distribution of trajectory endpoints in southern Sichuan in April, May and June, respectively; (**g**–**i**), Distribution of trajectory endpoints in western Sichuan in April, May and June, respectively; (**j**–**l**), Distribution of trajectory endpoints in central Sichuan in April, May and June, respectively; (**m**–**n**), Distribution of trajectory endpoints in eastern Sichuan in April and May; (**o**), Distribution of trajectory endpoints in northern Sichuan in June.

The spatial distribution of valid endpoints for the back trajectory from April to June indicated seasonal variation in the source areas of the migrating FAWs in Sichuan (Figure 3 and Table 1). In addition to local breeding populations, FAWs also migrated to southwestern Sichuan. The distribution of back trajectory endpoints from southwestern Sichuan was similar in April and May, both in northwestern Yunnan and northern Myanmar (Figure 3a,b). In June, the endpoints were concentrated in western Yunnan, with a few in the fringe of northeastern Yunnan, but a few were also located in Myanmar (Figure 3c). The endpoints from southern Sichuan were broader than those from southwestern Sichuan and were distributed in western Guizhou and the northern part of Yunnan; in addition, some endpoints were in northern Guangxi, and the furthest endpoints were in the northern part of Myanmar during the month of April (Figure 3d). The endpoints in May were more concentrated than those in April and were primarily distributed in the junction area of the northeastern part of Yunnan and southern Sichuan, with the eastmost reaching northeastern Guizhou and the westmost extending into Myanmar (Figure 3e). In June, the endpoints had a wide range of components within the areas between 22°0’0’’ N and 33°0’0’’ N, mainly in central Sichuan, western Guizhou, central Yunnan, and western Chongqing, reaching as far north as northeastern Sichuan and as far south as northern Vietnam (Figure 3f).

The trajectory endpoints from western Sichuan were concentrated in the year-round breeding areas of southwestern Sichuan in April (Figure 3g) and reached northwest Yunnan in May (Figure 3h). The endpoints extended further north and east into central and eastern Sichuan and Chongqing in June (Figure 3i). Trajectories originating in eastern Sichuan were terminated mainly in northern and western Guizhou and eastern Yunnan, with a few endpoints also in southwestern Sichuan in April (Figure 3m). In May, the endpoints were widespread and shifted more eastward, mainly in Guizhou and Yunnan, and as far east as northwestern Hunan (Figure 3n). The endpoints from central Sichuan were distributed in the northern and northwestern parts of Guizhou and a few in eastern Yunnan in April (Figure 3j). There were few valid endpoints in May, and the endpoints were concentrated along the border of northern Guizhou and Chongqing (Figure 3k). By contrast, in June, the endpoints were widely distributed, mainly in Chongqing, eastern Sichuan, southern Sichuan, Guizhou, northwestern Guangxi, and eastern Yunnan (Figure 3l). FAWs migrated into northern Sichuan in June, with endpoints distributed in the east, mainly in eastern and central Sichuan, Chongqing, and a few in Guizhou (Figure 3o).

From April to June, the potential source areas of the FAWs varied monthly and expanded northward and eastward. The potential source areas of the FAWs in Sichuan were concentrated in Yunnan, Guizhou, Chongqing, and Myanmar of the western pathway of national migration and were also located in Vietnam, Guangxi, and Hunan of the eastern pathway. At various times, some parts of Sichuan have also served as source areas for other parts of Sichuan.

### 3.3. Wind Field Analysis during FAW Migration

**Figure 4:**
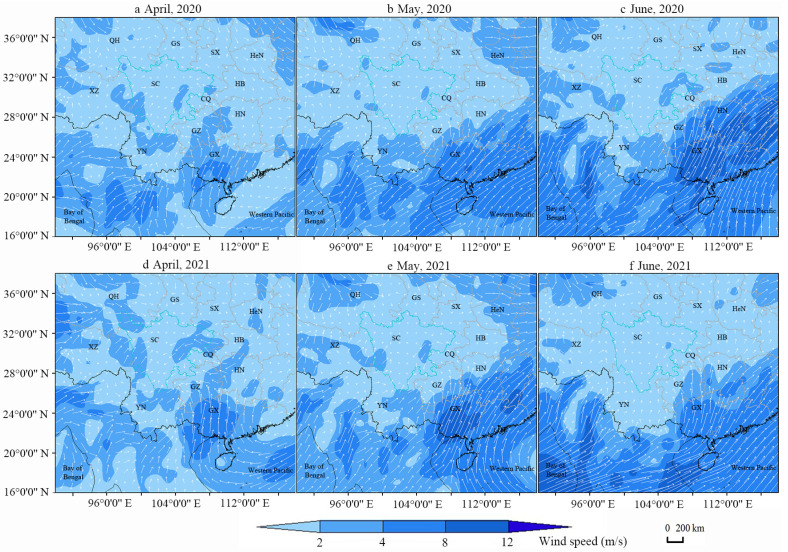
Average wind field at 850 hPa from April to June in 2020 and 2021. The white arrow shows the wind direction and wind speed. The longer and denser the arrows are, the higher the wind speed. The blue color shows the wind speed. Two-letter codes refer to the different provinces of China (SC: Sichuan Province, YN: Yunnan Province, GX: Guangxi Zhuang Autonomous Region, GZ: Guizhou Province, CQ: Municipality of Chongqing, HN: Hunan Province, HB: Hubei Province, HeN: Henan Province, SX: Shaanxi Province, GS: Gansu Province, XZ: Tibet Autonomous Region, QH: Qinghai Province). (**a**–**c**), Average wind field at 850 hPa in April, May and June 2020, respectively; (**d**–**f**), Average wind field at 850 hPa in April, May and June 2021, respectively.

In April, the prevailing west-southwesterly winds in Myanmar and Yunnan and southerly-southeasterly airflows in Guizhou provided the carrier airflow for the FAWs to migrate into the Sichuan Province (Figure 4a,d). Thus, the source areas were mostly located to the south and southwest of the starting point of the back trajectories. In May, the center of the subtropical high moved northward, and Yunnan and southern China were controlled by the southwest wind. The southwest airflow traveled to Guizhou and Hunan before gradually turning into a south-southeast airflow, which provided the carrier airflow for the FAWs to migrate from Yunnan, Guizhou, Hunan, and other places to Sichuan; the insect source also moved westward and northward accordingly (Figure 4b,e). In June, the southwestern airflow further controlled Yunnan and southern China, providing the necessary airflow for the FAWs to move from Yunnan to southwest Sichuan. The southwest airflow that traveled to Guizhou and Chongqing gradually turned into south and west winds and developed into a cyclone over the Sichuan Basin (Figure 4c,f). Therefore, the FAWs in the Sichuan Basin came not only from the southwest and south but also from the west and northeast, migrating from Yunnan, Guizhou, Chongqing, and the rest of Sichuan.

### 3.4. Migratory Pathways of FAW

**Figure 5:**
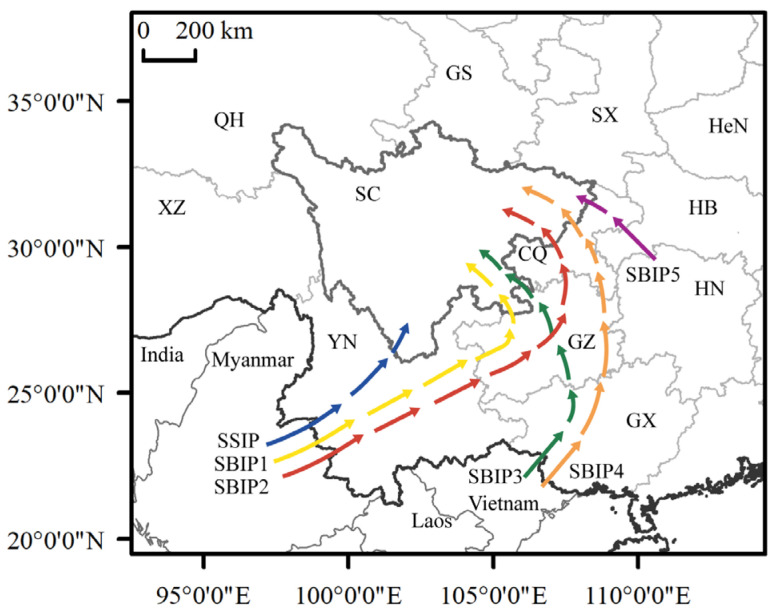
The FAW migratory pathways to Sichuan. There are 6 potential migratory pathways to Sichuan, 1 southwest Sichuan migratory pathway and 5 Sichuan Basin migratory pathways. SSIP: Southwest Sichuan migratory (Blue arrow). SBIP1: Sichuan Basin migratory pathway 1 (yellow arrow). SBIP2: Sichuan Basin migratory pathway 2 (red arrow). SBIP3: Sichuan Basin migratory pathway 3 (green arrow). SBIP4: Sichuan Basin migratory pathway 4 (orange arrow). SBIP5: Sichuan Basin migratory pathway 5 (purple arrow). The two-letter codes refer to the different provinces of China (SC: Sichuan Province, YN: Yunnan Province, GX: Guangxi Zhuang Autonomous Region, GZ: Guizhou Province, CQ: Municipality of Chongqing, HN: Hunan Province, HB: Hubei Province, HeN: Henan Province, SX: Shaanxi Province, GS: Gansu Province, XZ: Tibet Autonomous Region, QH: Qinghai Province).

The potential migration paths of the FAW were analyzed based on the immigration trajectory and the horizontal wind field at 850 hPa during the migration period. There were 6 potential migratory pathways into Sichuan, 1 southwest Sichuan migratory pathway and 5 Sichuan Basin migratory pathways (Figure 5). The southwest Sichuan migratory pathway was mainly from Myanmar through Yunnan or directly from Yunnan to southwest Sichuan (SSIP). The Sichuan Basin migratory pathways were relatively scattered. Pathway 1: Myanmar—Yunnan—Guizhou—Sichuan Basin (SBIP1); Pathway 2: Myanmar—Yunnan—Guizhou—Chongqing—Sichuan Basin (SBIP2); Pathway 3: Vietnam—Guangxi—Guizhou—Sichuan Basin (SBIP3); Pathway 4: Vietnam—Guangxi—Guizhou—Chongqing—Sichuan Basin (SBIP4); Pathway 5: Hunan-Chongqing-Sichuan Basin (SBIP5). Meanwhile, all the provinces and municipalities along the five paths could have provided insect sources for Sichuan at different times; however, pathways 1 and 2 were the main migratory pathways to the Sichuan Basin.

## 4. Discussion

In this study, we simulated the source areas and the migratory pathways of FAWs in Sichuan using a trajectory approach that considered both the flight behavior parameters of the FAW and the background weather conditions. Since 2020 and 2021 were the second and third years of FAW invasion in Sichuan, there may also be overwintering insect sources in some areas. However, Jiang et al. studied the winter breeding and overwintering areas of FAW in China, and pointed out that the overwintering area in Sichuan may have only provided a limited spring insect sources and that the main source of FAW in spring was in the immigrant population [12]. Meanwhile, there were no field survey data in recent years showing that FAWs overwintered in the potential overwintering area of Sichuan. Therefore, this paper assumes that the first discovery of FAW in Sichuan was the immigrant population. Compared with 2019, the first year of FAW migration into Sichuan, the arrival time of FAWs into some areas of Sichuan in 2020 and 2021 occurred earlier, and the source area distribution in 2020 and 2021 was further to the north and east. This may be because after invading China, FAWs bred year-round in tropical and subtropical areas such as Yunnan, Guangxi, Guangdong, and Hainan; thus, they formed a stable and closer insect source for migration to the northern areas [12], which could provide for earlier migrations. The results showed that despite the differences between 2020 and 2021, the general trend of the FAW migration into Sichuan was the same, with FAWs migrating into Sichuan sporadically in April and on a large scale in May before spreading throughout the entire Sichuan Basin in June.

According to previous studies, FAWs migrate from south to north through two migratory pathways: the eastern and western pathways. Sichuan is located on the western migration pathway in China [11]. The migratory populations of FAWs along the two pathways were relatively separated, and only a few individuals of the west migratory population migrated eastward into the Hunan, Hubei, and Henan Provinces, where they mixed with the populations from the eastern pathway [11,15]. Our study, on the other hand, showed that the FAW source areas in Sichuan were widely distributed. In addition, Myanmar, Yunnan, Guizhou, and Chongqing were on the western migration pathway, while Vietnam, Guangxi, Hunan, and Hubei were on the eastern migration pathway. Therefore, there may be frequent exchanges between the FAW populations in the eastern and western pathways, and Sichuan is an important node for population exchange.

Our study showed that the migration trend in Sichuan was from south to north and from east to west, which was different from the two FAW migratory pathways in China, both from southwest to northeast [11,15]. Insects are small and have a limited ability to fly, and their seasonal long-distance migration is directly affected by the atmospheric environment [36,37,38]. Two tropical ocean heat sources and water vapor sources in the South China Sea and the Bay of Bengal jointly influenced the FAW southwest-to-northeast migration in eastern China during spring and summer [13]. This southwest wind also carried FAWs from Myanmar and Yunnan to southwestern and southern Sichuan. However, affected by the unique topography of the Qinghai-Tibet Plateau, the southwest wind gradually turned into south and west winds in Guizhou and Chongqing and formed a cyclone over the Sichuan Basin, which transported FAWs from east to west, even from the northeast.

The migration trend of FAWs was also slightly different from another migratory pests in Sichuan, such as the white-backed planthopper, *Sogatella furcifera* (Horváth), which can be found from southeast to northwest [16,18,20]. In addition to migration from abroad, the FAW breeds year-round in Yunnan and parts of southwestern Sichuan and can overwinter in parts of southern Sichuan [22], so Southwest Sichuan is the place where FAWs first appear in Sichuan every year. *S*. *furcifera* can only overwinter in a few places in southern Hainan and the southernmost part of Yunnan. The main source of *S*. *furcifera* in China during the spring is from abroad [21]. Every year, *S*. *furcifera* migrate into southern Sichuan, including Luzhou, on a large scale during late April [19] while sporadically migrating into southwestern Sichuan in May [18]. The difference between the first appearance period of the two pests in southwestern and southern Sichuan may be one of the reasons for the different migratory trends in Sichuan.

Most airborne insects migrate downwind [39]. However, some airborne insects do not completely drift with the wind, and their flight behaviors involve a certain degree of initiative. For example, *Spodoptera exigua* and *Helicoverpa armigera*—other noctuid moths—are capable of orientation and can aggregate into layers when migrating [40,41]. These behaviors can enable insects to achieve rapid and efficient migration and increase the success rate of their migration [42]. However, until now, there have been no direct observational data about the flight behavior of FAW moths in Asia, so the orientation of the FAW was not considered, and multiple flight altitudes were set to ensure that the most likely flight altitudes were captured. In this study, the valid trajectories were distributed across all altitudes between 500 and 1500 m, with similar proportions, which were different from Yunnan and Guizhou [11]. In previous studies, FAW could migrate hundreds of kilometers a night at the right wind speeds [4]. Similarly, in this study, we found that the longest migration distance of FAW was more than 1100 km in the potential migration pathways.

Based on our results, in addition to monitoring and controlling FAWs in year-round breeding areas and overwintering areas, the Sichuan Province should be aware of the FAW migration that starts in April. From May to June, FAW monitoring and prevention should be carried out across the whole province according to the insect source situations in the surrounding provinces. Moreover, the aerial flight behavior of FAWs in southern and southwestern China should be further studied by radar observation, and the landing mechanism of FAWs should be conducted in combination with meteorological conditions in order to develop accurate early warning systems and the scientific control of FAWs.

## Figures and Tables

**Table 1:** The distribution probability of the FAW backward trajectory endpoints in different regions of Sichuan Province from April to June.

Regions	Source Areas					
	April		May		June	
	Main Source Areas	Other Source Areas	Main Source Areas	Other Source Areas	Main Source Areas	Other Source Areas
southwestern Sichuan	Yunnan (59.60%) Myanmar (40.40%)	—	Yunnan (77.20%) Myanmar (22.80%)	—	Yunnan (97.60%)	Myanmar (2.40%)
southern Sichuan	Guizhou (51.41%) Yunnan (36.06%)	Myanmar (8.44%) Guangxi (4.09%)	Yunnan (62.07%) Guizhou (24.83%) Myanmar (13.10%)	—	central Sichuan (32.39%) Guizhou (20.45%) Yunnan (16.80%) Chongqing (15.38%)	eastern Sichuan (8.10%) Guangxi (3.44%) Vietnam (3.44%)
western Sichuan	southwestern Sichuan (97.74%)	Yunnan (2.26%)	southwestern Sichuan (72.40%) Yunnan (20.89%)	Myanmar (4.99%) southern Sichuan (1.05%) Guizhou (0.66%)	central Sichuan (53.33%) Chongqing (24.91%) eastern Sichuan (21.75%)	—
central Sichuan	Guizhou (77.78%)Yunnan (22.22%)	—	the border of northern Guizhou and Chongqing (100%)	—	Chongqing (49.45%) eastern Sichuan (23.19%) Guizhou (19.01%)	southern Sichuan (5.93%) Guangxi (1.54%) Yunnan (0.88%)
eastern Sichuan	Guizhou (76.60%) Yunnan (17.02%)	southwestern Sichuan (6.38%)	Guizhou (49.05%) Hunan (31.33%) Yunnan (15.82%)	Guangxi (2.53%) Vietnam (1.27%)	—	—
northern Sichuan	—	—	—	—	eastern Sichuan (41.30%) central Sichuan (33.00%) Chongqing (19.43%)	Guizhou (6.07%) Guangxi (0.20%)

## Data Availability

The data presented in this study are available in article and Supplementary Materials.

## Supplementary Materials

The following supporting information can be downloaded at: https://www.mdpi.com/article/10.3390/insects13100935/s1, Table S1: The larva discovery date and estimated migration date of *Spodoptera frugiperda* in Sichuan Province in 2020; Table S2: The larva discovery date and estimated migration date of *Spodoptera frugiperda* in Sichuan Province in 2021.
