# Supplementary material for: The Source Areas and Migratory Pathways of the Fall Armyworm Spodoptera frugiperda (Smith) in Sichuan Province, China
Source: Insects. 2022 Oct 16;13(10):935. doi: 10.3390/insects13100935 (PMC9604329; doi:10.3390/insects13100935)
Supplement: Supplementary file 1 [file insects-13-00935-s001.zip › insects-1955843-supplementary.pdf]

Table S1. The larva discovery date and estimated migration date of *Spodoptera frugiperda* in Sichuan Province in 2020.

Time	Regions	Sites	Latitude/°N	Longitude/°E	Larva discovery date	Estimated immigration date
April	Southern Sichuan	Gulin County, Luzhou City	27.9792	105.9265	4.30	4.16-4.18
	Western Sichuan	Hanyuan County, Ya'an City	29.3502	102.6586	5.13	4.28-4.30
	Central Sichuan	Gaoping District, Nanchong City	30.7853	106.1296	5.7	4.23-4.25
	Eastern Sichuan	Dachuan District, Dazhou City	31.1960	107.5397	4.29	4.15-4.17
May	Southern Sichuan	Fushun County, Zigong City	29.1474	105.0132	5.21	5.7-5.9
	Western Sichuan	Shimian County, Ya'an City	29.2290	102.2836	6.1	5.18-5.20
		Emeishan City	29.5037	103.3841	6.4	5.21-5.23
	Central Sichuan	Yingshan County, Nanchong City	31.1587	106.7094	5.27	5.13-5.15
	Eastern Sichuan	Enyang District, Bazhong City	31.8276	106.5896	5.20	5.6-5.8
		Yuechi County, Guang'an City	30.5334	106.4117	6.4	5.21-5.23
June	Southern Sichuan	Gongxian county, Yibin City	28.1921	104.7959	6.22	6.8-6.10
	Western Sichuan	Jintang County, Chengdu City	30.7231	104.6074	6.28	6.14-6.16
	Central Sichuan	Shehong City	30.8662	105.3331	6.19	6.5-6.7
		Dongxing District, Neijiang City	29.6181	105.0638	6.25	6.11-6.13
	Northern Sichuan	Lizhou District, Guangyuan City	32.4319	105.9404	6.18	6.4-6.6
		Chaotian District, Guangyuan City	32.6427	105.9202	6.29	6.15-6.17

Table S2 The larva discovery date and estimated migration date of *Spodoptera frugiperda* in Sichuan Province in 2021

Time	Regions	Sites	Latitude/°N	Longitude/°E	Larva discovery date	Estimated immigration date
April	Southwestern Sichuan	Xichang City, Liangshan Prefecture	27.9006	102.2715	4.12	4.3-4.5
		Huili County, Liangshan Prefecture	26.6570	102.2082	4.21	4.12-4.14
		Leibo County, Liangshan Prefecture	28.2690	103.5784	4.23	4.16-4.18
	Southern Sichuan	Xuyong County, Luzhou City	28.1618	105.4512	4.23	4.14-4.16
May	Southwestern Sichuan	Yanyuan County, Liangshan Prefecture	27.4282	101.5154	5.11	5.4-5.6
		Meigu County, Liangshan Prefecture	28.3340	103.1386	5.18	5.14-5.15
		Mianning County, Liangshan Prefecture	28.5553	102.1836	5.31	5.24-5.26
	Southern Sichuan	Gaoxian County, Yibin City	28.4425	104.5244	5.18	5.8-5.10
	Western Sichuan	Mabian Yi Autonomous County, Leshan City	28.8417	103.5525	5.17	5.13-5.14
		Shimian County, Ya'an City	29.3502	102.6586	6.4	5.26-5.28
	Eastern Sichuan	Dazhu County, Dazhou City	30.7421	107.2116	5.7	5.3-5.4
		Dachuan District, Dazhou City	31.2024	107.5184	5.19	5.12-5.14
June	Southwestern Sichuan	Dechang County, Liangshan Prefecture	27.4085	102.1826	6.7	6.1-6.2
		Zhaojue County, Liangshan Prefecture	28.0205	102.8494	6.22	6.15-6.17
	Southern Sichuan	Weiyuan County, Neijiang City	29.5336	104.6745	6.11	6.6-6.7
		Rongxian County, Zigong City	29.4510	104.4239	6.16	6.7-6.9
		Luxian County, Luzhou City	29.1578	105.3883	6.25	6.14-6.16
	Western Sichuan	Jintang County, Chengdu	30.8684	104.4184	7.6	6.29-6.30
	Central Sichuan	Nanbu County, Nanchong City	31.3537	106.0436	6.16	6.11-6.12
		Pengxi County, Suining City	30.7638	105.7134	6.24	6.15-6.17
	Northern Sichuan	Lizhou District, Guangyuan City	32.4402	105.8524	6.17	6.10-6.12
		Jiange County, Guangyuan City	32.2935	105.5316	7.7	6.28-6.30
